# Differences in gut microbiota among patients with anastomotic leak following colorectal cancer surgery

**DOI:** 10.3389/fmicb.2025.1578990

**Published:** 2025-10-06

**Authors:** Junfeng Yan, Qiushuang Wang, Qiang Li, Jiatong Lu, Qiang Tong

**Affiliations:** Department of Gastrointestinal Surgery I Section, Renmin Hospital of Wuhan University, Wuhan, China

**Keywords:** colorectal cancer, anastomotic leak, gut microbiota, 16S rRNA sequencing, colorectal surgery

## Abstract

**Background:**

Anastomotic leak (AL) continues to be one of the most severe complications following colorectal surgery, with its incidence showing little change over time. The causes of anastomotic leak are varied, and the precise underlying mechanisms remain elusive. However, there is a growing body of evidence indicating a significant association between the intestinal microbiota and the occurrence of anastomotic leak.

**Methods:**

This study aimed to investigate the preoperative gut microbiota in patients who developed anastomotic leak (AL) following colorectal surgery. We collected preoperative fecal samples from both patients who developed anastomotic leak and those who did not for clinical research. Utilizing 16S Ribosomal RNA (16S rRNA) sequencing, we examined differences in gut microbiota of preoperative patients with colorectal cancer to identify bacterial species that may either mitigate or exacerbate the risk of anastomotic leak. Additionally, we preoperatively fed mice with *Lactobacillus case*i Zhang to demonstrate its protective effects against anastomotic leak.

**Results:**

We observed a significant increase in the diversity of intestinal microbiota in patients with anastomotic leak compared to those without. Specifically, the populations of *Lachnospiraceae* (*p* < 0.05), *Bacteroidaceae* (*p* < 0.01), and *Fusobacteriaceae* (*p* < 0.05) were markedly higher in patients with anastomotic leak, whereas *Lactobacillaceae* levels were comparatively lower (*p* < 0.05). Animal studies further supported these findings; mice preoperatively fed with *Lactobacillus* demonstrated a significantly reduced likelihood of developing anastomotic leak compared to those on a normal diet, with a statistical significance (*P* < 0.05).

**Conclusion:**

This study indicates that the presence of *Lachnospiraceae*, *Bacteroidaceae*, and *Fusobacteriaceae* in the intestinal microbiota may contribute to the development of anastomotic leak. Conversely, *Lactobacillus* appears to serve a protective role, potentially inhibiting this complication. Animal experiments further support these findings, demonstrating that preoperative supplementation with *Lactobacillus* may effectively prevent anastomotic leak. Our research may offer a novel preventive strategy for preoperative prevention of anastomotic leak.

## Introduction

Postoperative anastomotic leak (AL) remains one of the most severe and consequential complications of colorectal surgery. It is associated with increased specific mortality from tumors and both local and distant tumor recurrences, which contribute to a decline in the 5-year relative and disease-free survival rates for patients (Nachiappan and Faiz, 2015; Lu et al., 2016; Arron et al., 2022). Despite advancements in surgical techniques, including the use of laparoscopy and automated suturing devices over recent decades, the global incidence rate of postoperative anastomotic leak still ranges from 3 to 15% (Zimmermann et al., 2019; Crippa et al., 2020). The factors influencing the occurrence of anastomotic leak are multifaceted, potentially involving surgical procedures, blood flow, tension at the anastomosis site, and inflammation, among others, necessitating further research to fully elucidate the specific causes (Kang et al., 2013; Frasson et al., 2015; Reisinger et al., 2015).

The human gastrointestinal tract houses a vast array of microbes, whose collective communities, environments, and metabolites comprise the gut microbiome. This microbiome plays a crucial role in the healing process of intestinal anastomoses. Early research, dating back to 1994, indicated that a normal gut flora could promote wound healing (Okada, 1994). Subsequent studies, including those by Swanson and others, have demonstrated that natural symbiotic bacteria stimulate the production of reactive oxygen species (ROS) in intestinal epithelia, which inactivates adhesion kinase phosphatase and promotes epithelial cell repair (Swanson K. et al., 2011). Further research has shown that *Fusobacterium nucleatum* in the gut microbiome can induce colonic anastomotic leak by activating MMP9 expression in epithelial cells (Shi L. et al., 2022). Animal experiments suggest that a normal gut microbiome, compared to germ-free conditions or a single microbial species, enhances the healing of intestinal anastomoses (Okada and Kanazawa, 1999). Additionally, certain pathogens like *Enterococcus faecalis* may contribute to anastomotic leak by producing collagenase or activating host metalloproteinases that degrade collagen in the intestinal tissue (Shogan et al., 2015). The gut microbiome also influences anastomotic healing through the modulation of pro-inflammatory cytokines in the mucosa (Hajjar G. et al., 2023). Studies examining postoperative anastomotic tissues in rats have noted significant increases in *Enterococcus* and *Escherichia* coli at the injury site (Shogan et al., 2014).

Recent advances in microbiome research have further elucidated the gut microbiota’s involvement in postoperative complications, including anastomotic leak. Hajjar R. et al. (2023) demonstrated that certain preoperative microbial signatures–particularly elevated levels of *Alistipes onderdonkii* and reduced levels of *Parabacteroides goldsteinii*–were significantly associated with the risk of anastomotic leak in colorectal cancer patients, likely due to their respective pro-inflammatory and protective roles in mucosal healing. In a murine model, Boatman et al. (2023) showed that diet-induced shifts in gut microbial composition could influence anastomotic healing, where Western-style diets promoted dysbiosis and impaired healing, while high-fiber diets enriched with *Lactobacillus* species improved outcomes.

Additionally, emerging evidence suggests that surgical bowel preparation and perioperative antibiotic use significantly impact the gut microbial balance, which may in turn influence wound healing capacity. Recent reviews have highlighted that interventions such as preoperative fiber supplementation or short-chain fatty acid (SCFA) administration may enhance intestinal barrier function and reduce postoperative complications (Hajjar et al., 2024).

Furthermore, a recent Danish cohort study demonstrated that the baseline fecal microbiome, sampled weeks before surgery, differed significantly between patients who developed anastomotic leak and those who did not, with the AL-group showing an overrepresentation of collagenase-producing bacteria prior to surgery Cell (Jørgensen et al., 2024). A 2024 scoping review of both clinical and experimental studies further corroborated these observations, identifying reduced α-diversity and consistent enrichment of genera such as *Lachnospiraceae*, *Bacteroidaceae*, *Fusobacterium*, and *Bifidobacterium* in AL cases. In contrast, genera including *Prevotella* and *Eubacterium* appeared to be protective (Lianos et al., 2024).

Mechanistic insights from surgical infection literature emphasize that pathogenic organisms like *Enterococcus faecalis* and *Pseudomonas aeruginosa* produce collagenases and activate host metalloproteinases at the anastomotic site. Their abundance is often triggered by perioperative factors (e.g., antibiotics, Western diet), promoting a dysbiotic “pathobiome” that increases AL risk (Chang and Guyton, 2023). Moreover, the MIRACLe pilot study (2022) showed that a protocol combining oral antibiotics, mechanical bowel preparation, and perioperative probiotics reduced the rate of AL from 6.4 to 1.7% in elective laparoscopic colorectal resections, suggesting microbiota modulation may be a viable prevention strategy (Carlini et al., 2022).

While there is increasing evidence of the gut microbiome’s significant impact on anastomotic healing, current research, primarily derived from animal models, has yet to definitively characterize the microbiome composition in surgical patients and its link to anastomotic leak. Therefore, this study aims to determine the gut microbial composition of patients with anastomotic leak in comparison to general patients, identifying potential beneficial and harmful bacterial species (Hajjar and Dagbert, 2019).

In this study, we found that patients with anastomotic leak exhibit a significantly higher bacterial diversity compared to normal patients. Notably, there were increases in the abundance of *Lachnospiraceae*, Bacteroidaceae and *Fusobacteriaceae*, while the presence of the *Lactobacillus* phylum was diminished. These findings suggest that alterations in the gut microbiome may directly contribute to the development of anastomotic leak. Further research is needed to elucidate both the promotive and protective roles of these bacteria in relation to anastomotic leak.

## Materials and methods

### Patients and clinical samples

This study was conducted at Hubei Provincial People’s Hospital and included colorectal cancer patients undergoing colorectal resection. All patients underwent left-sided colorectal resections, including low anterior resection and sigmoid colectomy. Exclusion criteria included patients under 18 years of age, those undergoing emergency surgeries, and those who had received neoadjuvant radiotherapy or chemotherapy prior to surgery, and those whose fecal samples were not collected in a timely manner before the operation. All patients received mechanical bowel preparation and oral antibiotic therapy according to the standard protocol of Hubei Provincial People’s Hospital (Polyethylene glycol electrolyte solution was administered on the day before surgery, combined with oral cefuroxime 1 *g* and metronidazole 1 *g*). Anastomotic leak were diagnosed using the Diagnostic Score (DIACOLE) (Rojas-Machado et al., 2016). Fecal samples were collected from 200 patients on the morning before surgery, prior to the administration of any antibiotics or bowel preparation. Among them, 10 patients developed anastomotic leakage postoperatively. Using the Propensity Score Matching (PSM) method (PSM was performed based on age, gender, BMI, tumor location, albumin level, prophylactic stoma, and presence of diabetes) (Rosenbaum and Rubin, 1983), we selected 10 corresponding patients who did not develop anastomotic leak. We then performed 16S rRNA gene sequencing on fecal samples from these 20 patients. This study was approved by the Ethics Committee of Renmin Hospital of Wuhan University (Approval No. WDRY2018-K055), and written informed consent was obtained from all patients.

### 16S rRNA gene sequencing and bioinformatics analysis

16S rRNA gene sequencing and bioinformatics analysis were performed on the microbial DNA extracted from fecal samples using the TRIzol^®^ reagent (Invitrogen) following the manufacturer’s guidelines. The V3-V4 hypervariable regions of the bacterial 16S rRNA gene were amplified, and the purified amplicons were pooled at equimolar concentrations for sequencing. 16S rRNA Gene Sequencing analysis was performed using BMKCloud^1^. We used the normalized relative abundance matrix to identify significantly different microbial features between groups using the Wilcoxon rank-sum test. OTUs were clustered at 97% similarity using the QIIME pipeline. Beta diversity was evaluated using Bray–Curtis distance and visualized through PCoA. The significance of group differences was assessed using ANOSIM and PERMANOVA with 999 permutations. The Wilcoxon rank-sum test was used for differential abundance, adjusted by Benjamini–Hochberg FDR. The clustering patterns of microbial profiles, measured by Bray-Curtis distance, were analyzed using ANOSIM and betadisper tests, each with 999 permutations. LEfSe (Linear Discriminant Analysis Effect Size) analysis was employed to identify bacterial taxa that were significantly different between the two groups (patients with and without anastomotic leak). The analysis was performed using the default parameters of the LEfSe tool, with a threshold of LDA score ≥2 to determine the effect size of discriminative features. The significance of each taxon was assessed by using the Kruskal-Wallis test for inter-group comparison followed by pairwise Wilcoxon rank-sum tests. A threshold of *p* < 0.05 was used to identify taxa with significant differences between the groups.

### Mouse model

All experimental animals were 10–12 weeks old male C57BL/6 mice, sourced from Charles River Laboratories (Raleigh, North Carolina, USA). They were housed according to the Institutional Animal Care and Use Committee (IACUC) guidelines of the University of Chicago (IACUC Protocol 72417). The mice were maintained under standard laboratory conditions in filter-topped cages, acclimatized for 48 h in a temperature-controlled room (22–24 °C) with a 12-h light-dark cycle, and had free access to food and water. We divided sixty mice into two dietary groups: one receiving a *Lactobacillus casei* Zhang suspension (1 × 10^9^ CFU/200 μL) via oral gavage daily for 4 weeks, and the other receiving an equivalent volume of sterile saline. The *Lactobacillus casei* Zhang strain was originally isolated from traditional fermented koumiss and has been previously validated for probiotic activity.

Prior to surgery, mice underwent a low anterior resection of the rectum as described in prior studies (Hajjar et al., 2024; Jørgensen et al., 2024). Anesthesia was administered 30 min before surgery via an intraperitoneal injection of ketamine (100 mg/kg) and xylazine (10 mg/kg). We performed the surgery using a sterile abdominal midline incision, transecting the colon, and the anastomosis was completed using interrupted 6-0 polypropylene sutures. After the surgery, all animals received a subcutaneous injection of 1 ml of 0.9% saline for recovery. Carprofen (5 mg/kg) was given subcutaneously once daily for 3 days for postoperative pain management.

One week post-operation, we euthanized the mice to evaluate anastomotic healing. The integrity of the anastomosis was verified by inflating the distal colon with saline during an enema. We assessed anastomotic healing using the Anastomotic Healing Score (AHS) from previous studies: AHS 0 represented normal healing; AHS 1, fragile adhesion; AHS 2, dense adhesions without abscess; AHS 3, dense adhesions with a visible abscess; and AHS 4, severe leak accompanied by peritonitis (Hajjar et al., 2024; Jørgensen et al., 2024). An AHS score of ≥3 was indicative of an anastomotic leak. All animals were randomly allocated to groups. Investigators assessing AHS were blinded to treatment.

### Statistical analysis

All categorical data were presented as case counts and percentages, and continuous data were presented as means ± standard deviation (SD) or medians with ranges. We used the Pearson Chi-square test to compare categorical data, while continuous data were analyzed using the independent sample *t*-test or the Mann-Whitney U test, depending on data distribution. Logistic regression models were utilized to identify independent risk factors for outcomes. Statistical analyses were conducted using SPSS version 23.0 and Prism 8.0 software. Statistical significance was defined as *p < 0.05, **p < 0.01, ***p < 0.001, and ****p < 0.0001.

## Results

Compared to the general patient population, those with anastomotic leaks exhibited a significant increase in gut microbiome diversity.

**FIGURE 1:**
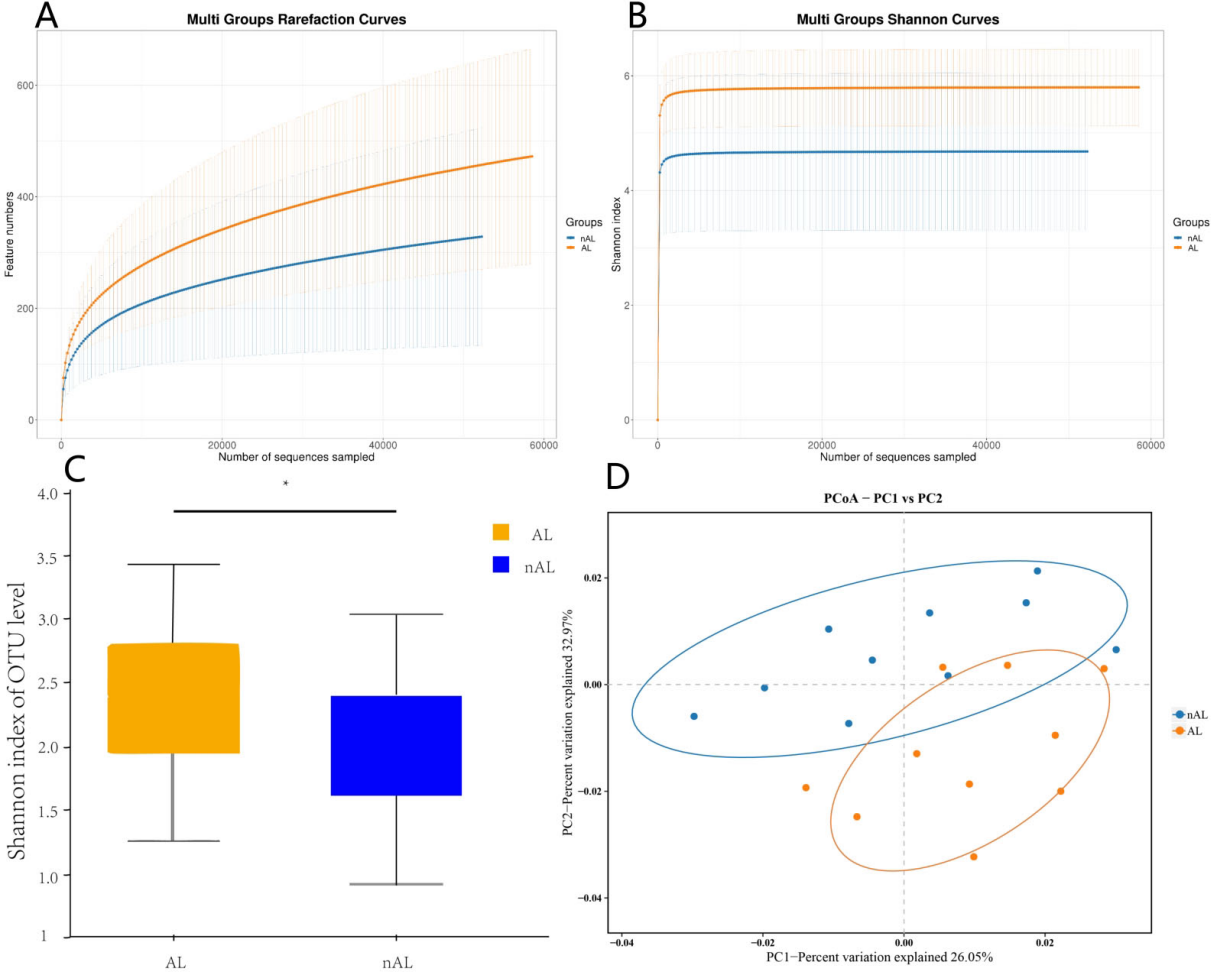
AL patients exhibit higher abundance of microbiota. **(A)** Rarefaction curves of species richness between the AL and nAL group. **(B,C)** Alpha diversity (Shannon index) between AL and nAL group. **(D)** The PCoA analyses based on BrayCurtis distance were used to reveal the β-diversity between AL and nAL group, ANOSIM statistical analysis.

To explore the potential relationship between the gut microbiome and anastomotic leak, we collected samples from 10 patients with anastomotic leak and 10 matched patients without anastomotic leak, aiming to minimize the influence of other risk factors (Table 1). Anastomotic leak was diagnosed at a median of 5 days postoperatively (range, 4–7 days). According to the International Study Group of Rectal Cancer (ISREC) criteria, 6 cases were categorized as Grade B and 4 cases as Grade C (Kulu et al., 2013). We analyzed the gut microbiome using 16S rRNA sequencing. Significant differences were observed in the Shannon indices at the OTU level between the two groups (Figures 1B, C). Principal Coordinates Analysis (PCoA) further revealed significant differences in β-diversity between the groups (Figure 1D).β-disper analysis confirmed that the significant ANOSIM results were not due to a non-uniform distribution of samples across groups.

**TABLE 1:** Patient clinical information.

Variable	Group nAL (*n* = 10)	Group AL (*n* = 10)	*p*
Age	61.1 ± 6.24	61.7 ± 6.09	0.830
Gender			1.000
Male	5 (50%)	5 (50%)	
Female	5 (50%)	5 (50%)	
BMI	23.83 ± 1.82	23.73 ± 2.06	0.916
Tumor height from anus	8 (5,8)	6 (5,8)	0.170
Albumin	42.06 ± 2.89	41.76 ± 3.08	0.823
Combined diabetes			1.000
No	8 (80%)	8 (80%)	
Yes	2 (20%)	2 (20%)	
Prophylactic stoma	2 (20%)	3 (20%)	

In patients with anastomotic leak, there was a notable increase in the abundance of *Lachnospiraceae*,*Bacteroidaceae* and *Fusobacteriaceae* accompanied by a relative decrease in *Lactobacillaceae*.

**FIGURE 2:**
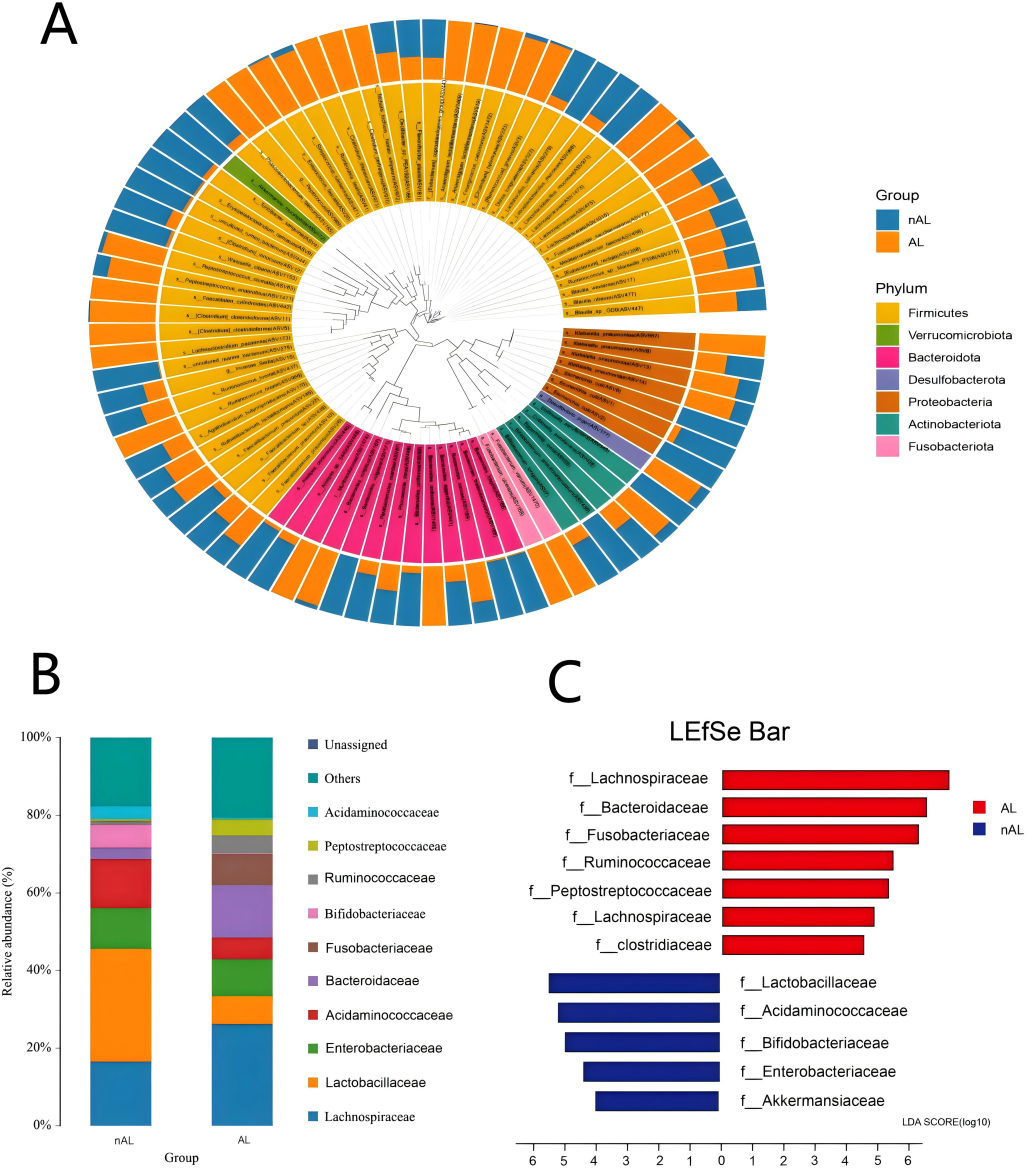
Differences in bacterial species between AL and nAL patients. **(A)** Sample community distribution results of AL and nAL patient species evolutionary tree (Phylum level). **(B)** The relative abundance of microbiota between AL and nAL group (family level). **(C)** LEfSe algorithm identifies differential bacteria between AL and nAL patients (family level).

The LEfSe method was employed to discern significant differences in species between the groups. As illustrated in Figure 2A, the relative abundance of various bacterial species in patients with anastomotic leak was contrasted with that of general patients. Specifically, *Lachnospiraceae*,*Bacteroidaceae* and *Fusobacteriaceae* exhibited a significant increase in abundance among patients with anastomotic leak, whereas *Lactobacillaceae* was more prevalent in normal patients (Figures 2B, C).

Preoperative administration of *Lactobacillus* effectively mitigated the occurrence of anastomotic leaks in mice.

**FIGURE 3:**
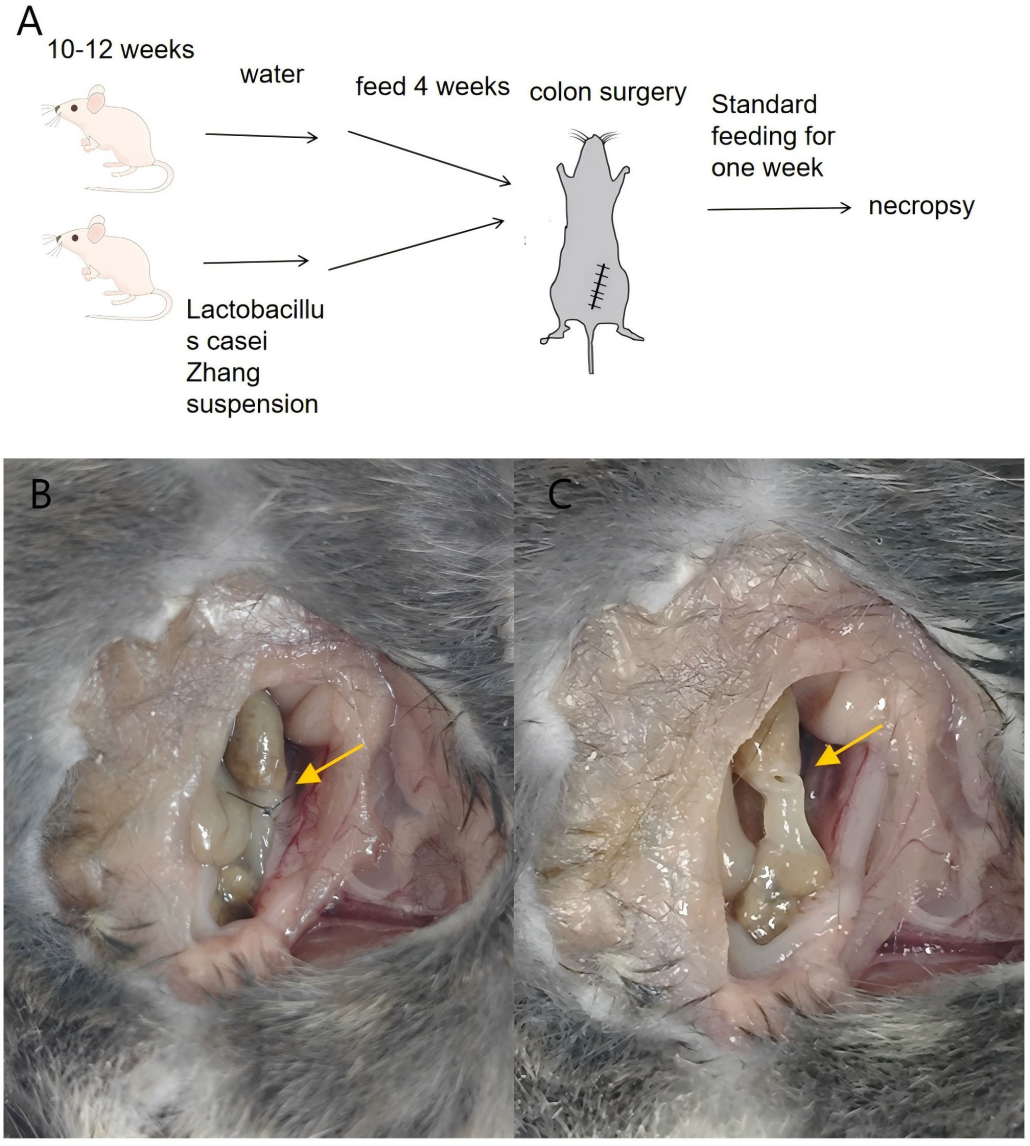
Preoperative oral administration of Lactobacillus casei Zhang reduces anastomotic leak in mice. (A) Schematic diagram of the anastomosis model. Mice were randomly divided into two groups: the treatment group (Lactobacillus administration group) and the control group. (B,C) Examination of anastomotic healing. figure B shows a normally healed intestinal anastomosis, while figure C illustrates an anastomosis with leak.

Among the 60 mice included in the study, 58 (95%) survived until necropsy, with no statistically significant difference in survival rates observed between the two groups (*P* = 1, Chi-square test). An Anastomotic Healing Score (AHS) of ≥3 was considered indicative of an anastomotic leak (Figure 3). In contrast, only 1 out of 29 mice in the *Lactobacillus casei* Zhang treatment group showed clinical leak, the incidence of anastomotic leaks was significantly reduced in the *Lactobacillus* group (*P* = 0.044, Chi-square test, see Table 2).

**TABLE 2:** The probability of anastomotic leak in mice treated with *lactobacillus* was significantly reduced.

Group	NoAL (n, %)	AL (n, %)	Total (n)	χ^2^	*P*-value
A (Lactobacillus)	28 (96.55)	1 (3.45)	29	4.062	0.044*
2-7 B (Control)	23 (79.31)	6 (20.69)	29		
All	51 (87.93)	7 (12.07)	58		

*p < 0.05.

Group A, *lactobacillus* treatment group; Group B, control group.

## Discussion

Postoperative anastomotic leak remains one of the most serious complications of colorectal cancer surgery, with a relatively high incidence worldwide. Despite advances in surgical techniques, the occurrence of anastomotic leak in certain patients appears almost inevitable. Complicating matters, the diverse presentations of anastomotic leak, coupled with the low specificity and sensitivity of these manifestations, often render early diagnosis challenging (Wiegerinck et al., 2018). Our study holds potential significance in predicting the occurrence of anastomotic leak. For high-risk patients exhibiting an increased abundance of harmful bacteria, proactive measures can be implemented. These may include intraoperative adjustments to the surgical approach, placement of abdominal and anal drainage tubes, and consideration of preventive ostomy (Gaines et al., 2020).

Although reduced microbial diversity is commonly associated with dysbiosis and adverse outcomes in gastrointestinal disorders, such as inflammatory bowel disease (IBD), colorectal cancer, and post-operative complications (Manichanh et al., 2012; Gao et al., 2015), some studies have reported that certain pathological states can exhibit increased diversity.

This paradoxical increase may reflect an overgrowth of pathogenic or opportunistic bacteria, contributing to intestinal barrier disruption. Our findings are align with these observations, suggesting that increased diversity may not necessarily indicate a healthy microbial state in the context of colorectal surgery. Similar patterns have been described by Swanson P. et al. (2011), who noted that oxidative stress–driven epithelial responses can be modulated by diverse microbial signals, and by Hajjar R. et al. (2023), who found altered pro-inflammatory responses associated with microbial changes despite elevated diversity levels.

While our study identified associations between specific bacterial taxa and the occurrence of anastomotic leak, the underlying biological mechanisms remain to be elucidated. *Fusobacteriaceae* and *Bacteroidaceae* have both been implicated in promoting inflammation and tissue degradation in the gastrointestinal tract. Specifically, *Fusobacterium nucleatum*, a member of the *Fusobacteriaceae* family, has been shown to upregulate matrix metalloproteinases, such as MMP9, which contribute to the breakdown of collagen and other extracellular matrix components, thereby impairing tissue repair processes. Shi S. et al. (2022) demonstrated that *Fusobacterium nucleatum* can activate epithelial cells to express MMP9, thereby promoting colon anastomosis leak by inducing collagen degradation.

Similarly, *Bacteroidaceae*, particularly *Bacteroides fragilis*, has been shown to modulate the intestinal environment in a way that could enhance disease progression. Research by Liu et al. (2022) suggested that Bacteroides fragilis can contribute to the formation of anaerobic niches, which favor pathogen colonization and inflammatory responses, leading to compromised anastomotic healing. Furthermore, *Bacteroidaceae* has been linked to the induction of intestinal inflammation, which could further disrupt the healing process at the anastomotic site (Lakemeyer et al., 2025).

In contrast, *Lactobacillus*, particularly the strain *Lactobacillus casei* Zhang, has shown significant protective effects in promoting gut mucosal integrity and reducing inflammation. Probiotic strains such as *Lactobacillus* are known to maintain intestinal homeostasis by enhancing the epithelial barrier and modulating the host’s immune responses. In a study by Lao et al. (2024), it was found that *Lactobacillus casei* supplementation significantly reduced gut inflammation and accelerated recovery from intestinal surgery by promoting tight junction formation and regulating cytokine levels (Shapiro et al., 2023). These findings align with our observations that *Lactobacillus* may play a protective role in preventing anastomotic leaks by mitigating inflammation and promoting epithelial regeneration.

While our study did not directly investigate these pathways, the association between microbial taxa and anastomotic leak suggests that microbial imbalances, particularly an overgrowth of pathogenic bacteria like *Fusobacterium* and *Bacteroides*, may exacerbate inflammation and tissue degradation. Conversely, the use of probiotic strains like *Lactobacillus casei* Zhang may offer a therapeutic approach to mitigate these adverse effects. Future mechanistic studies are warranted to clarify the causal role of these microbes in anastomotic healing. Such studies could include investigating the expression of MMP9, tight junction proteins, and other biomarkers of epithelial integrity and inflammation in relation to specific bacterial species.

The use of preoperative bowel cleansers in colorectal cancer surgery has been a contentious issue. While proponents argue their benefits in bowel cleansing and facilitating surgical procedures, preoperative mechanical bowel preparation often disrupts the delicate balance of the gut microbiome, leading to dysbiosis. Research conducted by Drago et al. has demonstrated a significant decrease in the abundance of *Lactobacillus* following bowel cleansing (Chen, 2021). *Lactobacillus*, known for its protective role, may play a crucial part in mitigating the risk of anastomotic leak, as suggested by our study’s findings (Zhao et al., 2022). Moreover, the use of bowel cleansers exerts a significant, albeit short-term, impact on the composition and diversity of fecal and gut microbiota, creating a conducive environment for pathogen colonization (Drago et al., 2016). Therefore, our research advocates for a reconsideration of the routine use of bowel cleansers before surgery.

The use of antibiotics represents another influential factor in the alteration of the gut microbiome. Antibiotic application has made significant strides in the realm of colorectal cancer surgery, with current consensus supporting the use of preoperative oral antibiotics to diminish postoperative complications and expedite hospital recovery (Gan et al., 2002; Shobar et al., 2016). However, certain antibiotics have been implicated in inducing bacterial translocation, which can provoke heightened inflammatory responses. Excessive inflammation, in turn, may precipitate a range of postoperative complications, underscoring the importance of judicious antibiotic use to prevent misuse and mitigate potential adverse outcomes (Morris et al., 2015).

Diet represents another pivotal factor influencing the intestinal microbiota, offering distinct advantages over bowel preparation and preoperative antibiotics. Unlike the rapid and transient effects of bowel preparation and the enduring impact of antibiotics, dietary changes exert a rapid and reproducible influence on the intestinal microbiota (David et al., 2014; Bachmann et al., 2017). Animal studies have underscored the significance of long-term dietary habits, demonstrating that a high-fat Western diet escalates the risk of anastomotic leaks, whereas a short-term preoperative regimen featuring low-fat, high-fiber intake can mitigate this risk. Alterations in diet have been shown to modulate the intestinal microbiota in mice, with a notable increase in *Lactobacillus* abundance observed in those consuming a low-fat, high-fiber diet–findings consistent with our own research (Foppa et al., 2020; Boatman et al., 2023). Nonetheless, a paucity of studies exists on the nexus between patient diet, intestinal microbiota, and anastomotic leaks, necessitating more comprehensive sequencing studies to unravel the intricacies of intestinal microbiota changes following dietary modifications.

While our study sheds light on the role of the intestinal microbiota in the healing process of anastomosis, it is important to acknowledge certain limitations. Despite mounting evidence indicating the significance of the intestinal microbiota, anastomotic leak remains associated with various confounding factors such as age, gender, and tumor location. While our study sought to mitigate these influences, the potential correlation between these factors and the intestinal microbiota remains unexplored. Anastomotic leak likely arises from a multifaceted interplay of factors and biological processes, encompassing host genetics, intestinal microbiota composition, inflammation, and immune responses. Recognizing the complexity of this interaction, it’s evident that a singular alteration in the intestinal microbiota may not yield optimal preventive outcomes. Hence, personalized and diversified treatment approaches tailored to individual patient profiles are warranted. Furthermore, it’s essential to acknowledge that our research constitutes a single-center retrospective study, underscoring the need for future multi-center studies with larger datasets to validate our findings. Although animal studies have underscored the protective role of *Lactobacillus* in preventing anastomotic leak, prospective human studies are imperative to corroborate these findings.

In summary, our study revealed distinctions in the preoperative intestinal microbiota ecology between patients with anastomotic leak and those without. Animal experiments have corroborated that preoperative manipulation of the intestinal microbiota can mitigate the incidence of anastomotic leak. Consequently, our findings suggest that preoperative modification of the intestinal microbiota holds promise as a preventive measure against anastomotic leak.

## Data Availability

The datasets presented in this study are publicly available. This data can be found here: https://www.ncbi.nlm.nih.gov, accession number PRJNA1334249.

## Footnotes

1
www.biocloud.net
